# Toward multimodal integration of colorectal cancer and chronic kidney disease: transcriptomic modeling as a framework for the SIRIO study “Spatial radiomics and transcriptomics to the discovery of the cross-link between colon cancer and chronic kidney disease”

**DOI:** 10.2478/raon-2026-0026

**Published:** 2026-04-14

**Authors:** Roberta Fusco, Vincenza Granata, Andrea Belli, Alessandra F Perna, Giovambattista Capasso, Michele Caraglia, Ugo Pace, Paolo Delrio, Ludovico Docimo, Claudio Gambardella, Francesco Saverio Lucido, Matteo Floris, Giorgia Locci, Matteo Runfola, Denise Giannascoli, Martina Izzo, Eugenio Sorgente, Margherita Borriello, Francesco Izzo, Mariadelina Simeoni, Antonella Petrillo, SIRIO Trial Collaborators

**Affiliations:** 1 Division of Radiology, Istituto Nazionale Tumori IRCCS Fondazione Pascale - IRCCS di Napoli, Naples, Italy; 2 Division of Epatobiliary Surgical Oncology, Istituto Nazionale Tumori IRCCS Fondazione Pascale - IRCCS di Napoli, Naples, Italy; 3 Department of Translational Medical Science, University of Campania Luigi Vanvitelli, Naples, Italy; 4 Biogem Scarl, Avellino, Italy; 5 Department of Precision Medicine, University of Campania Luigi Vanvitelli, Naples, Italy; 6 Colorectal Surgical Oncology-Abdominal Oncology Department, Istituto Nazionale Tumori IRCCS Fondazione Pascale – IRCCS di Napoli, Naples, Italy; 7 Division of General, Oncological, Mini-invasive and Bariatric Surgery, Department of Advanced Medical and Surgical (DAMSS), University of Campania Luigi Vanvitelli, Naples, Italy; 8 Department of Medical Sciences and Public Health, University of Cagliari, Cagliari, Italy; 9 Nephrology, Dialysis and Transplantation, ARNAS Brotzu, Cagliari, Italy; 10 Unit of Anatomic Pathology, ARNAS Brotzu, Cagliari, Italy; 11 General and Emergency Surgery Unit, ARNAS Brotzu, Cagliari, Italy; * From Istituto Nazionale Tumori IRCCS Fondazione Pascale IRCCS di Napoli: Sergio Venanzio Setola, Igino Simonetti, Roberta Galdiero, Andrea Belli, Renato Patrone, Mauro Piccirillo, Carmen Cutolo, Silvia De Franciscis, Daniela Rega, Massimiliano Di Marzo, Luisa Sgariglia, Mariadora De Feo, Tersa Pagano, Maria D’Amico, Antonio Avallone, Antonio Cassata, Carmen Romano, Sabrina Rossetti, Marilena Di Napoli, Gerardo Ferrara, Anna Maria Anniciello, Ciro Cipullo, Rossella De Cecio, Florinda Feroce, Tiziana Salviato, Valeria Varone, Saverio Simonelli, Giovanna Pignata, Ernesta Cavalcanti, Pietro Marone, Mario de Bellis, Paolo Andreozzi, Monica Cantile, Giosuè Scognamiglio, From University of Campania Luigi Vanvitelli: Carminia Maria Della Corte; Cagliari,

**Keywords:** colorectal cancer, chronic kidney disease, transcriptomic, radiomics

## Abstract

**Background:**

Colorectal cancer (CRC) and chronic kidney disease (CKD) are major contributors to global morbidity and mortality. Increasing epidemiological and genetic evidence suggests a biologically plausible interplay between renal dysfunction and colorectal tumorigenesis. However, publicly available datasets rarely integrate structured renal function parameters with multi-omic cancer data, limiting mechanistic and prognostic investigations of the CRC-CKD axis.

**methods:**

We systematically evaluated public resources, including the cancer genome atlas (TCGA) - colon adenocarcinoma (COAD) and an open-access transcriptomic survival dataset, to assess the feasibility of integrating clinical and genomic information for biomarker discovery. Transcriptomic data from 62 CRC patients were analyzed using unsupervised clustering, correlation-based feature selection, and multiple supervised machine learning classifiers to identify gene signatures associated with disease-free survival (DFS).

**Results:**

TCGA-COAD confirmed the canonical mutational landscape of CRC but lacked structured renal function data. In contrast, the survival dataset enabled integrative DFS modelling. Unsupervised analysis identified three transcriptionally distinct subgroups. Random forest and logistic regression achieved the highest predictive performance. Feature importance analysis highlighted *CYP2E1, RAB39A*, and *ZBTB3* as top-ranked predictors of recurrence risk.

**Conclusions:**

Our findings expose a critical gap in current public repositories regarding integrated CRC-CKD data and demonstrate the feasibility of transcriptomic-driven prognostic modelling. This analysis provides a hypothesisgenerating computational framework to support future multimodal investigations integrating renal, molecular, and imaging parameters in CRC that will be the main objectives of the SIRIO study.

## Introduction

Chronic kidney disease (CKD) and colorectal cancer (CRC) represent two converging epidemics in modern medicine, both contributing substantially to global morbidity, mortality, and healthcare burden. CKD affects approximately 10–15% of the global population and remains one of the leading causes of death, particularly among aging individuals and patients with multiple comorbidities.^1^ Beyond its cardiovascular implications, CKD is increasingly recognized as a systemic disorder characterized by chronic inflammation, immune dysregulation, endothelial dysfunction, metabolic derangements, and accumulation of uremic toxins.

CRC ranks as the third most frequently diagnosed cancer and the second leading cause of cancer-related deaths worldwide, with more than 1.9 million new cases and nearly 904,000 deaths recorded in 2022. The global incidence is projected to rise to approximately 3.15 million new cases and 1.6 million deaths by 2040, underscoring its escalating public health impact.^2^ While established risk factors for CRC include age, diet, lifestyle, genetic predisposition, and inflammatory bowel diseases, emerging evidence suggests that chronic systemic conditions, particularly CKD, may significantly modulate colorectal carcinogenesis.

Importantly, cancer represents the leading cause of non-cardiovascular mortality among CKD patients. Multiple epidemiological studies have reported an increased incidence and mortality of various malignancies, including CRC, in this vulnerable population.^3^ The biological plausibility of this association is supported by CKD-related pathophysiological alterations. Chronic low-grade inflammation, oxidative stress, impaired DNA repair, dysbiosis, and reduced immune surveillance collectively create a pro-tumorigenic milieu.^4^ Persistent proteinuria, a surrogate marker of glomerular injury and systemic endothelial dysfunction, has been independently associated with elevated CRC risk in large-scale population-based cohorts, suggesting that renal damage may reflect or amplify systemic oncogenic processes.^4^

Recent high-level evidence further strengthens this association. A meta-analysis combined with Mendelian randomization (MR) conducted by Qian *et al*. (2025)^1^ provided genetic and epidemiological support for a causal relationship between CKD and CRC, demonstrating that CKD significantly increases CRC risk (RR = 1.332, 95% CI: 1.084–1.637; OR = 1.171, 95% CI: 1.063–1.289, p = 0.001). Notably, subgroup analyses revealed a more pronounced effect in younger individuals and women, suggesting that renal dysfunction may act as an underrecognized, biologically active modifier of colorectal carcinogenesis rather than a mere comorbidity.

From a clinical perspective, the CRC-CKD interplay extends beyond incidence. Patients with impaired renal function often experience altered drug metabolism, increased chemotherapy toxicity, limited eligibility for clinical trials, and sub-optimal oncologic management. Despite this, individuals with CKD are frequently underrepresented in oncologic studies, limiting evidence-based treatment strategies tailored to this subgroup. Consequently, CKD may influence not only cancer risk but also tumor biology, therapeutic response, recurrence patterns, and survival outcomes.

At the molecular level, renal dysfunction may reshape the tumor microenvironment (TME) through systemic inflammatory signaling, vascular remodeling, and metabolic reprogramming. Identifying transcriptomic, radiomic, and integrated biomarkers that capture this kidney-tumor axis could significantly improve recurrence risk stratification, enable earlier detection of aggressive phenotypes, and support precision oncology approaches in CRC patients with or without CKD.

However, despite growing biological and epidemiological interest in the CRC-CKD axis, publicly available datasets rarely provide integrated clinical, renal, transcriptomic, and imaging information. This fragmentation prevents comprehensive multi-omics exploration and hampers the identification of clinically actionable patterns linking renal dysfunction to colorectal tumor behavior. The absence of structured renal function data (e.g, estimated glomerular filtration rate [eGFR], creatinine levels, CKD staging) within major oncologic repositories represents a critical knowledge gap.

To address this unmet need, our study was designed with a dual objective: first, to systematically identify and evaluate publicly accessible datasets suitable for exploring the CRC-CKD connection; and second, to implement a pilot integrative transcriptomic-machine learning workflow as a proof-of-concept framework.

Specifically, the main aims of this study are: (i) to assess the availability and limitations of public resources for investigating the CRC-CKD association, and (ii) to develop an integrated transcriptomic and machine learning pipeline as a preliminary model for future multimodal analyses.

Through the evaluation of public databases and the preliminary development of predictive machine learning pipelines, this study aims to define molecular and imaging biomarkers associated with CRC recurrence and progression. Furthermore, it lays the methodological foundation for the integrative multi-omics analysis of patients enrolled in the national PNRR-MCNT1-2023-12378005 SIRIO project.^5^ The PNRR-funded SIRIO project was established to generate a harmonized, prospective dataset integrating clinical, radiological, transcriptomic, and behavioral information in CRC patients, both with and without CKD. The project includes structured patient enrollment, tissue and liquid biopsy collection, CT imaging with radiomic feature extraction, and spatial and single-cell transcriptomic analyses to dissect the tumor microenvironment within a multimodal framework. By bridging nephrology and oncology through integrated data generation, the SIRIO study aims to elucidate the biological and clinical foundations of the CRC-CKD association and overcome the current limitations of fragmented public resources.

Currently available public datasets lack structured renal function variables such as eGFR, serum creatinine, or CKD staging, which prevents direct modeling of the biological interaction between CKD and CRC. Consequently, the present study focuses on colorectal cancer datasets to develop and validate a transcriptomic and machinelearning analytical framework. This framework is intended to serve as methodological groundwork for future integrative analyses and will ultimately be applied to the prospective SIRIO cohort, where renal function parameters will be systematically integrated with imaging and multi-omics data to investigate the CRC-CKD interplay.

## Patients and methods

### Identification of free access public databases

A systematic and structured search was conducted to identify publicly available datasets suitable for investigating the biological and clinical association between CRC and CKD. Databases were screened across multiple established biomedical and oncologic data repositories, including the Cancer genome atlas (TCGA); Genomic data commons (GDC); cBioPortal for Cancer Genomics; db-GaP; RadiomicsHub; Kaggle; The Cancer Imaging Archive (TCIA); and the UCSC Xena Browser.

The search strategy was designed to prioritize datasets enabling integrative and multi-domain analysis. Inclusion criteria were defined a priori and required: (i) availability of colorectal cancer patient data; (ii) presence of structured clinical annotations, including demographics, comorbidities, and survival outcomes; (iii) availability of transcriptomic or genomic data (e.g., RNA sequencing, microarray gene expression profiles, somatic mutation data); (iv) availability of imaging data, particularly contrast-enhanced computed tomography (CT) scans suitable for radiomic analysis; (v) explicit indicato of renal function or comorbid CKD (e.g., serum creatinine, eGFR, CKD staging, ICD coding); and (vi) open-access availability or controlled access through formal application procedures.

Particular attention was given to datasets potentially allowing cross-domain integration between renal function metrics and oncologic molecular profiling. Databases containing only single-domain information (e.g., genomics without renal parameters, or nephrology datasets without oncologic variables) were catalogued but critically appraised for their limitations in addressing the CRC-CKD interplay.

Importantly, databases providing raw imaging data without pre-extracted radiomic features were not excluded. Instead, they were retained for review when image quality, acquisition standardization, and DICOM availability permitted independent radiomic feature extraction. This approach ensured the identification of resources suitable for downstream multimodal integration, even when radiomics had not been previously computed.

### Selection of datasets for pilot analysis

Based on the screening results, two datasets were selected for exploratory computational analysis, chosen for their suitability in testing integrative transcriptomic and machine learning workflows within a CRC context.

TCGA-COAD (colon adenocarcinoma): This dataset includes comprehensive clinical annotations, RNA sequencing (RNA-seq) expression profiles, and somatic mutation data for more than 450 CRC patients. It is publicly accessible through the GDC portal.^6^ TCGA-COAD was selected to characterize the mutational landscape of CRC, explore transcriptomic patterns, and assess the feasibility of radiogenomic correlations. Although highly informative from an oncogenomic perspective, this dataset does not contain structured renal function data and was therefore not suitable for direct CRC-CKD integrative analysis. Nevertheless, it provided a robust reference framework for molecular stratification and downstream modeling. TCGA-COAD was therefore used primarily as a reference oncogenomic dataset to characterize the canonical molecular landscape of colorectal cancer and to test the analytical workflow. While it cannot provide direct insights into CKD-related mechanisms due to the absence of renal variables, it serves as a methodological foundation for future integrative analyses in datasets where renal function parameters are available.

CRC Gene Expression and Survival Dataset (GitHub): This open-access dataset comprises clinical and transcriptomic data from 62 CRC patients with curated disease-free survival (DFS) information.^7^ The dataset was used exclusively for transcriptomic DFS classification modeling and not for CKD linkage, as it does not include renal function variables. Two complementary files were integrated:

Clinical variables: age, sex, Dukes stage, treatment history, DFS time, and DFS event status.

Gene expression data: normalized microarraybased expression levels for 1,935 genes.

This dataset was selected due to the availability of survival endpoints, enabling supervised modeling of recurrence risk. It provided a suitable testbed to evaluate clustering strategies, feature selection methods, dimensionality reduction techniques, and multiple supervised classification algorithms in a controlled setting. Only patients with available gene expression profiles and complete clinical annotations, including DFS time and event status, were included in the analysis. Samples with missing survival information or incomplete transcriptomic data were excluded to ensure consistency of downstream modeling.

Together, these datasets were not intended to directly resolve the CRC-CKD association, but rather to: (i) characterize recurrent somatic mutations and transcriptional heterogeneity in CRC; (ii) assess the feasibility and robustness of integrative bioinformatic pipelines; and (iii) establish a methodological framework in preparation for the analysis of the prospectively collected multimodal SIRIO cohort, where renal function parameters will be systematically integrated with molecular and imaging data.

### Data processing

Sixteen public databases were identified for evaluation^7,10-24^, two of which were selected for further analysis: the TCGA-COAD dataset^24^ and the CRC Gene Expression and Survival dataset.^7^

The TCGA-COAD dataset^24^ was systematically evaluated to confirm the availability and integrity of structured clinical annotations and genomic data, including RNA sequencing (RNA-seq) expression profiles and somatic mutation calls. To characterize the molecular landscape of colorectal cancer (CRC), an exploratory mutational analysis was conducted focusing on the most recurrent somatic alterations. Mutation data were retrieved in tab-separated value (TSV) format and included detailed annotations such as mutation type (e.g., missense, stop gained, synonymous), affected gene and protein, variant classification, and the absolute number and percentage of patients harboring each mutation. Genes were prioritized based on both mutation frequency (absolute count) and cohort prevalence (percentage of affected patients), ensuring identification of biologically relevant driver events while minimizing noise from rare variants.

For the CRC Gene Expression and Survival dataset^7^, clinical and transcriptomic data tables were merged using unique patient identifiers. Gene expression values were standardized using z-score normalization across samples to ensure comparability and to prevent scale-driven bias in downstream modeling. Prior to modeling, exploratory data analysis was performed to assess distributional properties and identify potential outliers.

Clinical variables (e.g., age, tumor stage, DFS event) were encoded through binarization or categorical transformation where appropriate. Missing data were handled through complete case (case-wise) deletion to avoid imputation-induced bias in this small cohort. Given the limited sample size (n = 62) relative to feature dimensionality (1,935 genes), particular attention was paid to dimensionality reduction and overfitting mitigation.

The primary outcome variable for all predictive models was DFS event status, coded as 1 for recurrence and 0 for non-recurrence. To reduce dimensionality and limit the curse of dimensionality, feature selection was performed using correlationbased ranking with respect to DFS outcome. Only the subset of genes demonstrating the strongest positive or negative association with DFS was retained for downstream analysis. This step was designed to enhance signal-to-noise ratio and improve model interpretability. Gene prioritization was based on correlation ranking with respect to the DFS outcome rather than differential expression testing; therefore, multiple-testing correction procedures were not applicable.

Principal component analysis (PCA) was applied as an unsupervised dimensionality reduction technique to explore global transcriptomic variance structure, detect latent sample grouping, and visualize data distribution in two-dimensional space. PCA also allowed assessment of potential batch effects or dominant variance components unrelated to clinical outcome.

To investigate intrinsic molecular subgroups, unsupervised clustering was conducted using both K-means clustering and agglomerative hierarchical clustering with ward linkage, selected for their complementary partitioning strategies (centroid-based *vs*. variance-minimization hierarchical approach). The optimal number of clusters (k) was determined using multiple internal validation techniques^8^, including:

- Elbow method (evaluation of total within-cluster sum of squares).
- Silhouette score (mean silhouette coefficient across samples).
- Gap statistic (comparison between observed dispersion and reference null distribution).
- Concordance across these metrics was used to strengthen cluster robustness.

For supervised prediction of DFS events, five classification algorithms were implemented using the selected top-correlated genes as input features:

- Logistic regression (L2-regularized).
- Random forest.
- XGBoost.
- Decision tree.
- Gradient boosting.

To reduce model variance and prevent information leakage, the dataset was partitioned into 70% training and 30% testing subsets using stratified sampling to preserve class balance. All feature selection steps were performed within the training set to avoid optimistic bias. Additionally, 5-fold cross-validation was applied within the training cohort to assess model stability and generalizability.

Hyperparameters were tuned using cross-validated grid search where applicable. Given the small sample size, particular caution was taken to avoid overfitting, especially for high-capacity models (e.g., boosting-based methods). Model interpretability was assessed through feature importance analysis (for tree-based models) and coefficient magnitude (for linear models).

Model performance was evaluated using multiple complementary metrics^9^, including accuracy, precision, recall, F1-score, and area under the receiver operating characteristic curve (AUC). The use of both threshold-dependent (precision, recall, F1) and threshold-independent (AUC) metrics ensured a comprehensive assessment of discriminative performance, particularly relevant in moderately imbalanced survival outcome settings.

This structured computational workflow was conceived as a methodological proof-of-concept in preparation for the SIRIO cohort analysis, where renal function parameters (e.g., eGFR, creatinine, CKD staging) will be integrated with transcriptomic and radiomic features to enable multimodal modeling of CRC recurrence in patients with and without CKD.

All analyses were performed using publicly available datasets referenced in the manuscript, allowing independent reproduction of the workflow using R (version 4.4.2) within the RStudio environment. Data preprocessing, clustering analyses, and machine learning models were implemented using standard R packages. Random seeds were fixed where applicable to ensure reproducibility of the computational workflow.

The datasets used in this study are publicly available and referenced in the manuscript. Additional details regarding the computational workflow are available from the corresponding author upon reasonable request.

### Ethical consideration

This study used only publicly available, de-identified datasets and did not involve recruitment of human participants.

## Results

Despite the abundance of single-domain datasets, no publicly available dataset was found that included structured information simultaneously covering both CRC and CKD. In Table 1 was reported the evaluated public database.^7,10-24^

**TABLE 1.** Publicly available dataset with chronic kidney disease (CKD) or colorectal cancer (CRC) structured data

Dataset Name	Description	Use	Access	Link [reference]
Real colorectal cancer datasets from Kaggle	62 colorectal cancer patients and their respective gene expression levels.	Integration between clinical and transcriptomic data	Open access	[7]
UCI CKD dataset	400 patients, 24 clinical features	CKD stage classification	Open access	[10]
Kaggle CKD dataset	1,659 patients, clinical/lab data	Statistical analysis, ML	Open access	[11]
USRDS	National registry (CKD, ESRD)	Epidemiological studies	Request-based	[12]
UKRR CKD/AKI dataset	UK registry for CKD/AKI	Clinical audit, registry	Request *via* HDR UK	[13]
NHANES CKD	US survey with CKD diagnostics	Public health, epidemiology	Open access	[14]
dbGaP CKD genetics	Exome/genotype + phenotype	CKD genomics, GWAS	Request *via* dbGaP	[15]
PLCO CRC dataset	155,000 subjects, screening + followup	Screening, epidemiology	Request *via* CDAS	[16]
GENIE BPC CRC	1,485 patients, NGS + clinical	Precision medicine	Open access	[17]
TCIA CMB-CRC	Histopathology + radiology DICOM	Radiogenomics, AI	Open access	[18]
TCIA CRC_FFPE CODEX	35 multiplexed tumor images	Spatial biology, tumor microenvironment	Open access	[19]
ICCR CRC pathology	Standardized pathology templates	Diagnostic standardization	Open access	[20]
Kaggle CRC lifestyle	Diet, lifestyle, BMI, habits	Risk factor analysis	Open access	[21]
LC25000 & EBHI	25,000 histopath images in 5 classes	Image classification (CNN)	Open access	[22]
Kvasir-SEG	Polyp segmentation with masks	Endoscopic segmentation	Open access	[23]
TCGA-COAD	> 450 CRC patients, multi-omics	Oncogenomics, CMS	Open + dbGaP (raw)	[24]

1 AI = artificial Intelligence; AKI = acute kidney injury; CCN = convolutional neural network; CDAS = Cancer Data Access System; dbGaP = database of Genotypes and Phenotypes; CMS = Consensus Molecular Subtypes; DICOM = Digital Imaging and Communications in Medicine; EBHI = Enteroscope biopsy histopathological H&E image dataset; ESRD = end-stage renal disease; FFPE = formalin-fixed paraffin-embedded; GENIE BPC = Genomics Evidence Neoplasia Information Exchange Biopharma Collaborative; GWAS = genome-wide association study; HDR UK = health data research United Kingdom; ICCR = international collaboration cancer reporting; Kvasir-SEG = Segmented Polyp Dataset; LC25000 = Lung and colon histopathological image dataset, contains colour 25,000 images; ML = machine learning; NHANES = National Health and Nutrition Examination Survey; PLCO = Prostate, lung, colorectal and ovarian cancer screening trial; TCGA-COAD = the cancer genome atlas colon adenocarcinoma; TCIA CMB =The Cancer Imaging Arhiv Cancer Moonshot Biobank; UCI CKD = UC Irvin Machine Learning Repository chronic kidney disease; USRDS = United States Renal Data System data

The exploratory analysis of the somatic mutational landscape within the TCGA-COAD data-set^24^ identified Kirsten rat sarcoma viral oncogene homolog (KRAS) as the most frequently altered gene, with more than 12% of patients harboringthe canonical *KRAS G12D* variant. This hotspot mutation is a well-established driver alteration associated with constitutive activation of the RAS/MAPK signaling pathway and resistance to anti epidermal growth factor receptor (EGFR) therapies.

Additional high-frequency mutations were observed in adenomatous polyposis coli (APC), tumor protein p53 (TP53), PIK3CA, and SMAD4, all of which represent core components of colorectal tumorigenesis. APC mutations, typically early truncating events, are central to wingless-related integration site (WNT) pathway dysregulation and adenoma initiation. TP53 alterations contribute to genomic instability and tumor progression, whereas PIK3CA mutations activate the PI3K/AKT signaling axis, promoting proliferation and survival. SMAD4 inactivation disrupts TGF-β signaling and has been associated with metastatic potential and adverse prognosis.

Missense mutations represented the predominant type of genetic alteration across the cohort, reflecting selective pressure for functional protein changes rather than complete loss-of-function events. However, “stop gained” and synonymous variants were also detected, indicating a heterogeneous mutational spectrum. The predominance of missense mutations aligns with known CRC mutational patterns and reinforces the biological plausibility of the dataset.

Importantly, these findings are consistent with the established molecular taxonomy of colorectal cancer and validate the internal coherence of the TCGA-COAD dataset. The identification of recurrent driver mutations provides a robust genomic backbone for downstream analyses, including transcriptomic stratification and integrative modeling. Moreover, establishing the canonical mutational profile of the cohort was a necessary step to ensure biological interpretability prior to applying clustering and machine learning approaches.

**FIGURE 1.**
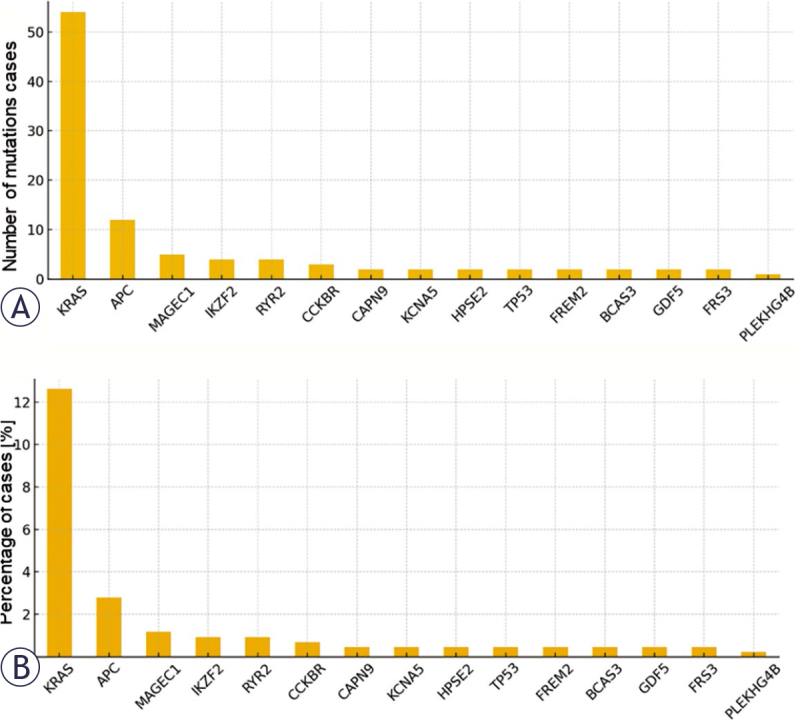
**(A)**Total number of mutated cases per gene. Bar plot representing the absolute number of patients in the cancer genome atlas colon adenocarcinoma (TCGA-COAD) cohort harboring somatic mutations in the most frequently altered genes. The top mutated genes (e.g., Kirsten rat sarcoma viral oncogene homolog [KRAS], adenomatous polyposis coli [APC], tumor protein p53 [TP53]) are displayed in descending order, highlighting their prevalence in colorectal cancer. **(B)** Cumulative percentage of patients with mutations per gene. Bar plot showing the cumulative percentage of patients affected by somatic mutations in each gene.

**FIGURE 2.**
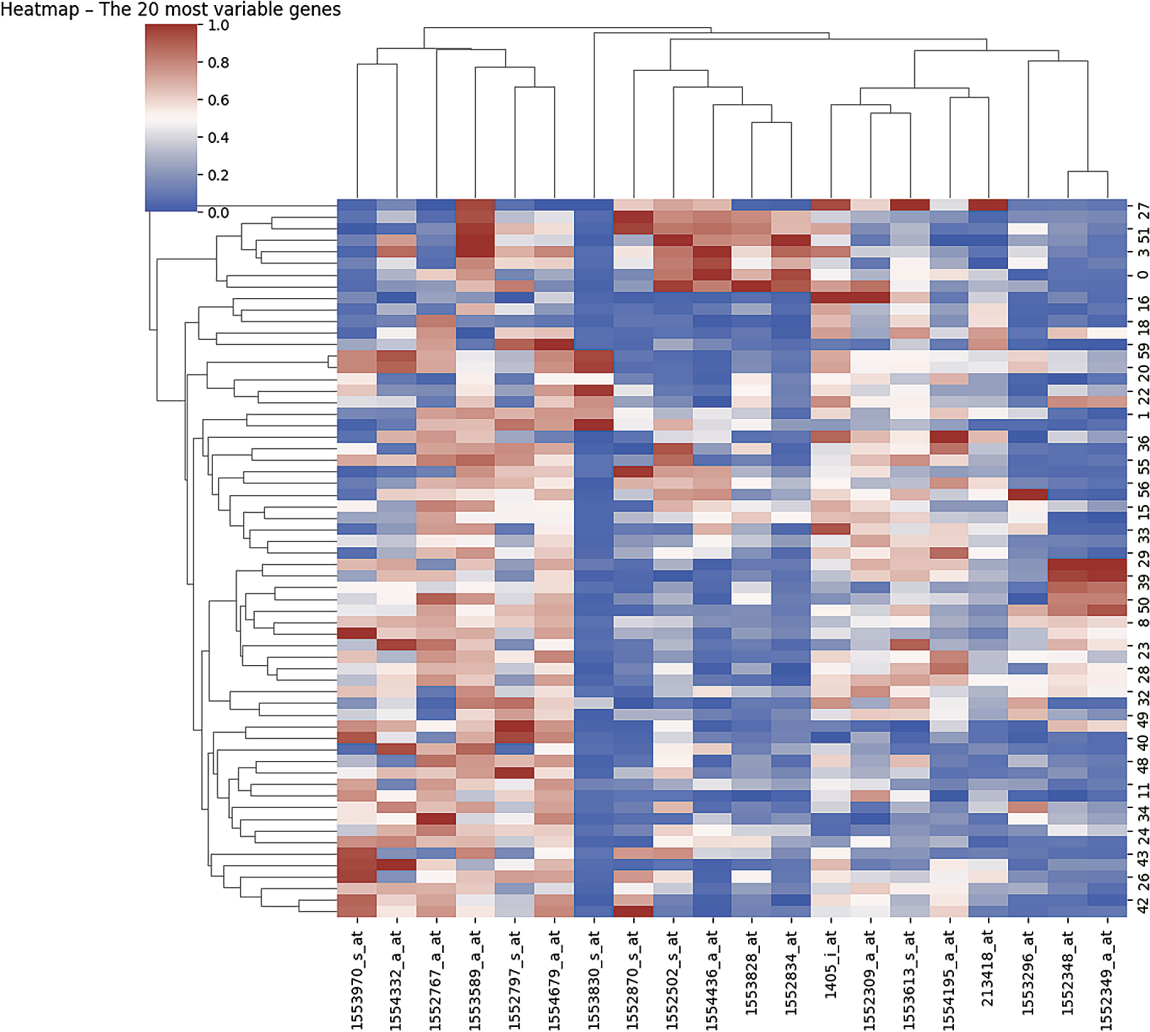
Heatmap of the top 20 most variable genes.

Summary visualizations of gene-level mutation frequencies and patient-level mutation prevalence are presented in Figure 1 and Figure 2.

The CRC Gene Expression and Survival dataset includes integrated clinical and transcriptomic data from 62 colorectal cancer (CRC) patients and is structured into two complementary files:

- CRC_SurvivalData.csv: containing patient identifiers (ID_REF), demographic variables (age, sex), Dukes tumor stage, tumor location (right *vs*. left colon), treatment history (radiotherapy, chemotherapy), DFS time expressed in months, and DFS event status (relapse or death).
- CRC_GeneExpression.csv: comprising normalized microarray-based gene expression values for 1,935 genes, matched to patient identifiers.

The cohort included 62 patients, with 37 DFS events (59.7%) and 25 non-events (40.3%). Given the relatively small sample size (n = 62), the predictive performance reported in this study should be interpreted cautiously and primarily as proof-of-concept. Larger independent cohorts will be required to confirm the robustness and generalizability of the proposed models.

Prior to analysis, gene expression data were standardized to ensure comparability across samples and to minimize scale-driven bias. Variancebased filtering was then applied to identify the most informative transcripts. A heatmap was generated to visualize the expression patterns of the top 20 most variable genes across all patients (Figure 2), as highly variable genes are more likely to capture biologically meaningful heterogeneity.

Hierarchical clustering was applied simultaneously to both genes and patient samples using Ward linkage and Euclidean distance metrics, enabling identification of co-expression modules and potential patient subgroups. The heatmap color scale ranged from red (high expression) to blue (low expression), with white indicating intermediate expression levels.

The accompanying dendrograms revealed structured clustering patterns, with coherent patient groupings and distinct gene co-expression signatures. These patterns suggest the presence of transcriptionally heterogeneous CRC subtypes within the cohort. The observed molecular stratification aligns with the established concept that CRC is not a single homogeneous entity but rather comprises biologically distinct subgroups with potentially different prognostic and therapeutic implications.

Importantly, this unsupervised visualization step served as an exploratory validation of intrinsic transcriptomic variability prior to supervised modeling. The identification of coherent expression clusters provided a rationale for subsequent dimensionality reduction, feature selection, and predictive classification analyses focused on DFS outcome.

**FIGURE 3.**
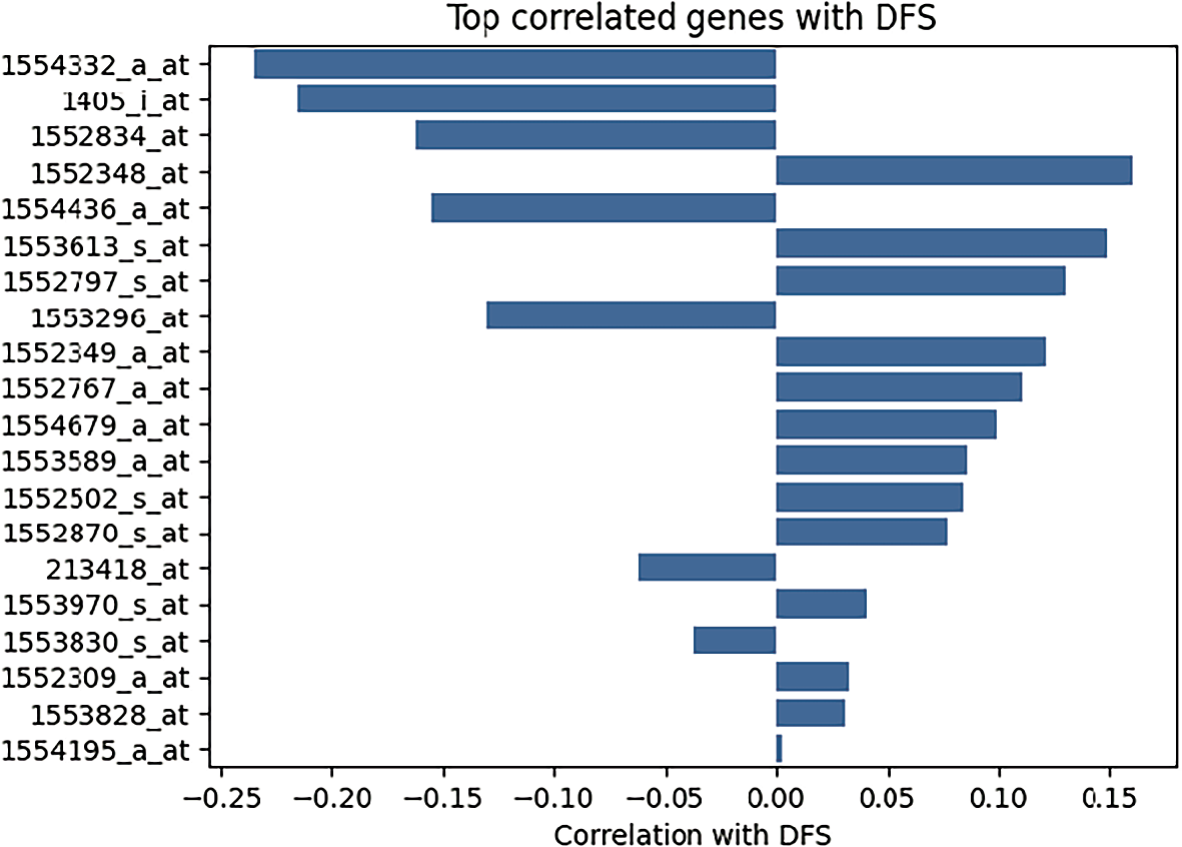
Bar plot top 20 correlated genes with disease-free survival (DFS).

The 20 genes showing the strongest positive or negative correlation with DFS were visualized in a dedicated bar plot (Figure 3), allowing a comparative overview of their relative association with recurrence risk.

Transcripts such as 1554332_a_at aldehyde dehydrogenase 1 family, member A1 (ALDH1A1) and 1405_i_at cyclin-dependent kinase 2 (CDK2) demonstrated negative correlations with DFS outcome, indicating that higher expression levels were associated with increased probability of recurrence. This suggests a potential role for these genes in promoting tumor aggressiveness or treatment resistance. ALDH1A1, in particular, has been implicated in cancer stem cell maintenance and chemoresistance, while CDK2 plays a central role in cell cycle progression and proliferative signaling.

Conversely, genes such as 1552348_at vascular endothelial growth factor A (VEGFA) and 1554436_a_at matrix metallopeptidase 9 (MMP9) exhibited positive correlations with DFS status. Depending on the coding scheme used (DFS event *vs*. DFS time), this pattern may indicate that elevated expression is associated with either improved disease-free interval or lower recurrence probability. Given the established biological functions of VEGFA (angiogenesis) and MMP9 (extracellular matrix remodeling and invasion), these findings warrant cautious interpretation and may reflect context-dependent effects or dataset-specific characteristics.

Overall, the correlation analysis highlights a subset of biologically relevant transcripts associated with recurrence dynamics. These genes were subsequently prioritized for clustering and supervised modeling, as they represent candidate molecular determinants of DFS heterogeneity within the cohort.

The selected genes (n = 20) were categorized according to their predominant biological functions to facilitate functional interpretation and pathwaylevel contextualization (Table 2). This grouping allowed a structured assessment of how distinct oncogenic processes may converge to influence DFS.

- **Oncogenes and tumor suppressors:***tumor protein p53 (TP53), Kirsten rat sarcoma viral oncogene homolog (KRAS), MYC proto-oncogene (MYC), Cyclin D1(CCND1), cyclin E1 (CCNE1)*
- These genes represent core regulators of cell cycle progression, genomic stability, and tumor initiation. TP53 loss impairs DNA damage response and apoptosis; KRAS mutations drive constitutive MAPK signaling; MYC regulates transcriptional amplification and metabolic re-programming; CCND1 and CCNE1 promote G1/S cell cycle transition. Their coordinated dysregulation reflects proliferative dominance and genomic instability – hallmarks of aggressive CRC phenotypes.
- **Growth factors and receptors:** *epidermal growth factor receptor (EGFR), human epidermal growth factor receptor 2 (HER2)*, v*ascular endothelial growth factor A* (VEGFA)
- These genes regulate proliferative and angiogenic signaling cascades. EGFR and HER2 activate downstream MAPK and PI3K/AKT pathways, while VEGFA orchestrates tumor angiogenesis and vascular remodeling. Overexpression of these factors may contribute to enhanced tumor growth, metastatic dissemination, and therapeutic resistance.
- **Intracellular signaling mediators:** *AKT serine/threonine kinase 1 (AKT1), signal transducer and activator of transcription 3 (STAT3), phosphatase and tensin homolog (PTEN)*
- These molecules modulate survival, apoptosis, and inflammatory signaling. AKT1 promotes cell survival and metabolic adaptation; STAT3 integrates inflammatory and oncogenic signaling; PTEN acts as a tumor suppressor by negatively regulating the PI3K/AKT axis. Alterations in this network may shift the balance toward sustained proliferative signaling and resistance to cell death.
- **Immune and inflammatory mediators:** *interleukin 6 (IL6), interleukin 10 (IL10), C-X-C motif chemokine ligand 8 (CXCL8), prostaglandin-endop eroxide synthase 2 (PTGS2)*
- These genes play pivotal roles in shaping the tumor microenvironment (TME). IL6 and CXCL8 are central mediators of chronic inflammation and may enhance tumor proliferation and immune evasion. PTGS2 (COX-2) contributes to prostaglandin-mediated inflammatory signaling and angiogenesis. IL10, traditionally antiinflammatory, may exert immunosuppressive effects within the TME, potentially facilitating tumor escape from immune surveillance.
- **Invasion and metastasis:** *matrix metallopeptidase 9 (MMP9)*
- MMP9 is a matrix metalloproteinase involved in extracellular matrix degradation, tumor invasion, and metastatic spread. Its expression reflects a more invasive phenotype and remodeling of the stromal microenvironment.
- **Detoxification and oxidative stress response:** *(aldehyde dehydrogenase 1 family, member A1) ALDH1A1*

**TABLE 2.** The description of the selected top 20 genes

Probe-set ID	Gene symbol	Gene title
1554332_a_at	*ALDH1A1*	Aldehyde dehydrogenase 1 family, member A1
1405_i_at	*CDK2*	Cyclin-dependent kinase 2
1552834_at	*EGFR*	Epidermal growth factor receptor
1552348_at	*VEGFA*	Vascular endothelial growth factor A
1554436_a_at	*MMP9*	Matrix metallopeptidase 9
1553613_s_at	*IL6*	Interleukin 6
1552797_s_at	*CXCL8*	C-X-C motif chemokine ligand 8
1553296_at	*STAT3*	Signal transducer and activator of transcription 3
1552349_a_at	*TP53*	Tumor protein p53
1552767_a_at	*CCND1*	Cyclin D1
1554679_a_at	*BCL2*	B-cell CLL/lymphoma 2
1553589_a_at	*AKT1*	AKT serine/threonine kinase 1
1552502_s_at	*PTEN*	Phosphatase and tensin homolog
1552870_s_at	*KRAS*	Kirsten rat sarcoma viral oncogene homolog
213418_at	*HER2*	Human epidermal growth factor receptor 2
1553970_s_at	*MYC*	MYC proto-oncogene, bHLH transcription factor
1553830_s_at	*CCNE1*	Cyclin E1
1552309_a_at	*CDKN1A*	Cyclin-dependent kinase inhibitor 1
1553828_at	*PTGS2*	Prostaglandin-endoperoxide synthase 2 (COX-2)
1554195_a_at	*IL10*	Interleukin 10

ALDH1A1 participates in aldehyde detoxification and oxidative stress modulation and has been associated with cancer stem cell characteristics, chemoresistance, and tumor recurrence.

Overall, this functional categorization underscores that the DFS-associated gene signature encompasses multiple hallmarks of cancer, including proliferation, angiogenesis, immune modulation, invasion, and metabolic adaptation. The convergence of these biological domains supports the hypothesis that recurrence risk in CRC is driven by coordinated dysregulation of interconnected oncogenic pathways rather than isolated molecular events.

**FIGURE 4.**
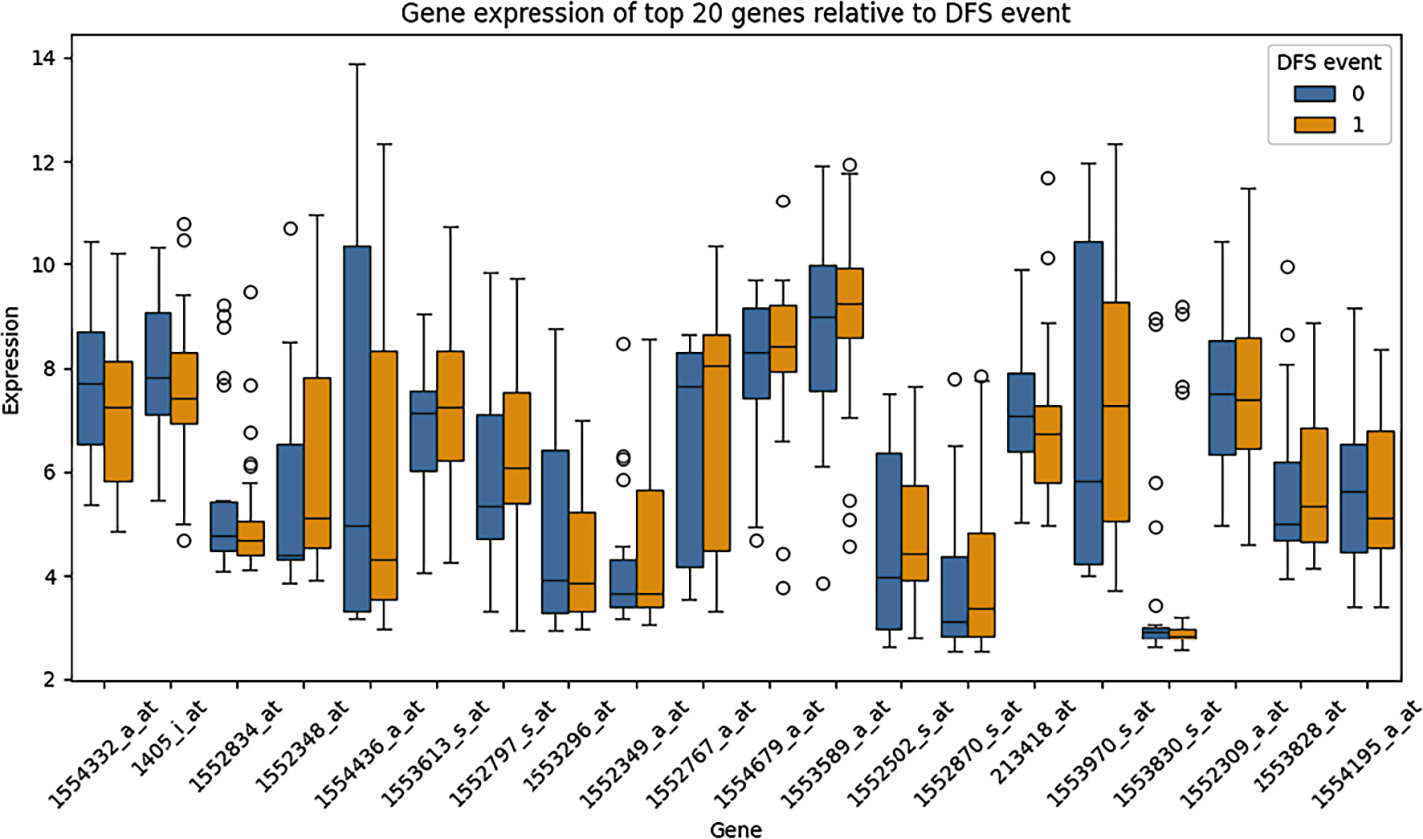
Boxplot of the top 20 correlated genes revealed group-wise differences between patients with and without disease-free survival (DFS) events.

Figure 4 illustrates the distribution of expression levels for the 20 transcripts most strongly correlated with DFS event status, stratified according to patient outcome (DFS event *vs*. no event). The boxplots provide a visual assessment of intergroup variability and reinforce the association between specific gene expression patterns and recurrence risk.

Notably:

- *ALDH1A1* (1554332_a_at) and CDK2 (1405_i_at) exhibited markedly lower expression levels in patients who experienced a DFS event compared with those who remained disease-free. This pattern suggests that higher expression of these genes may be associated with improved disease-free survival in this cohort. Although *CDK2* is classically linked to cell cycle progression, its context-dependent role and interaction with checkpoint regulators may influence tumor dynamics in a non-linear manner. Similarly, ALDH1A1, often associated with stemness and chemoresistance, may reflect subtype-specific biological behavior rather than uniformly adverse prognosis.
- In contrast, *VEGFA* (1552348_at) and matrix *MMP9* (1554436_a_at) showed increased expression in patients who experienced DFS events. This finding is biologically consistent with their established roles in tumor angiogenesis, extracellular matrix remodeling, invasion, and metastatic dissemination. Elevated *VEGFA* may promote neovascularization and tumor resilience, while *MMP*9 facilitates stromal degradation and tumor spread, supporting a more aggressive phenotype.

Overall, these expression differences reinforce the concept that recurrence risk in CRC is driven by coordinated dysregulation of angiogenic, invasive, and proliferative pathways. The observed intergroup variability further supports the bio-logical plausibility of the selected gene signature and justifies its use in subsequent clustering and supervised classification analyses.

**FIGURE 5.**
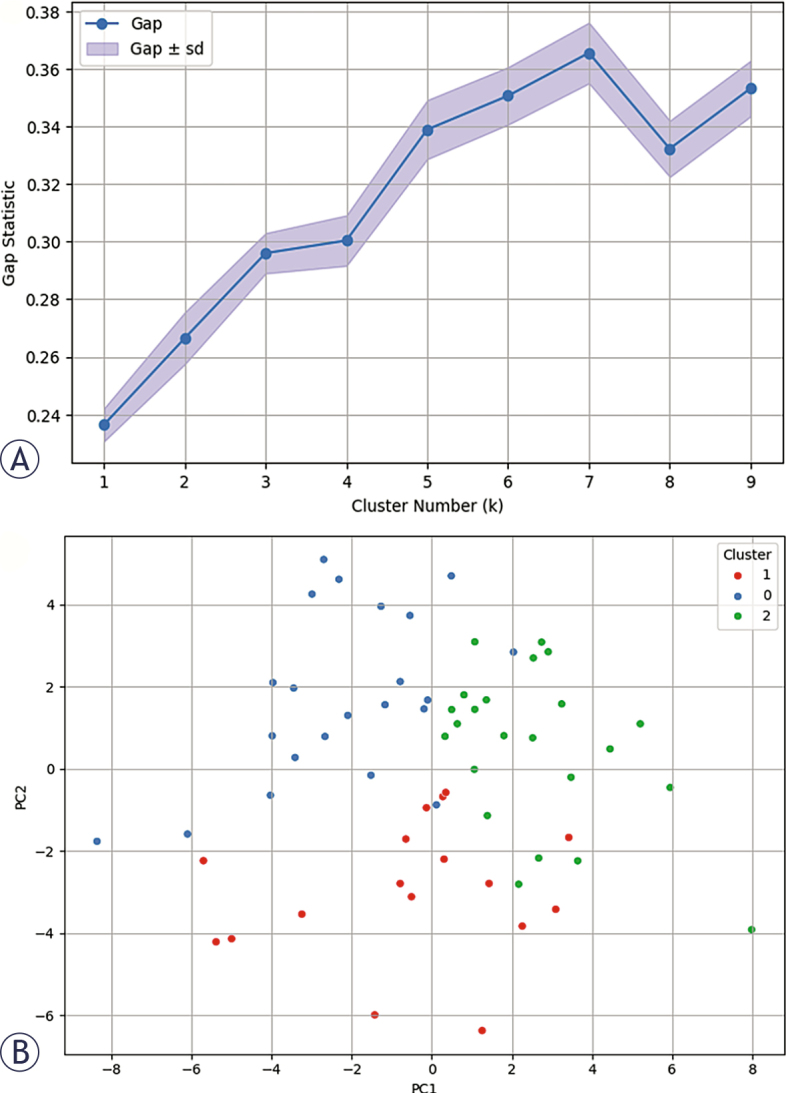
**(A)** Clustering with k = 3 (optimal value identified from gap statistic); **(B)** revealed three distinct patient clusters, visualized *via* principal component analysis (PCA) projection (Figure 5B)).

Final clustering with k = 3, identified as the optimal number of clusters based on the Gap Statistic, revealed three transcriptionally distinct patient groups (Figure 5). Concordance between the Gap Statistic and supportive trends observed in the Elbow method and Silhouette score strengthened the robustness of this partitioning strategy.

The three clusters were visualized through PCA projection onto a two-dimensional space, where separation between groups was clearly appreciable along the principal variance components. The spatial distribution of samples in PCA space suggests that the identified clusters are not random partitions but reflect structured transcriptomic heterogeneity within the cohort.

Importantly, the emergence of three distinct clusters supports the presence of intrinsic molecular subtypes characterized by differential gene expression programs. This stratification likely captures coordinated biological processes rather than isolated gene-level variations, reinforcing the hypothesis that CRC recurrence risk may be driven by subtype-specific transcriptional architectures.

Although derived from a relatively small cohort, the consistency between unsupervised clustering and dimensionality reduction visualization provides preliminary evidence of biologically meaningful subgrouping. These findings establish a rationale for subsequent comparative analyses across clusters and for integrating subtype information into supervised predictive modeling.

**FIGURE 6.**
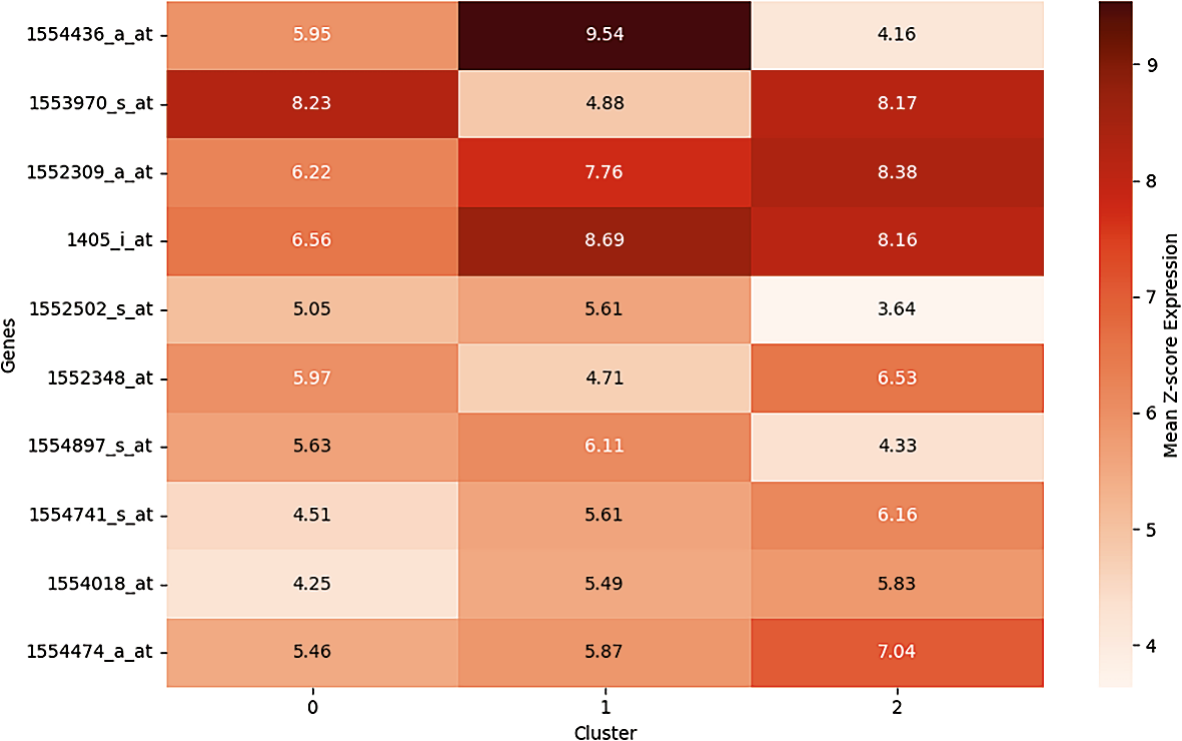
Top 10 Most variable genes across 3 clusters.

To further characterize the transcriptional programs underlying each molecular subgroup, we computed the mean expression levels of the top 10 most variable genes across the three clusters identified by K-means clustering. The resulting heatmap (Figure 6) highlights distinct gene expression signatures, underscoring structured biological heterogeneity among patient subgroups rather than stochastic variation.

Cluster 1 was characterized by elevated expression of *MMP9* (1554436_a_at), a matrix metalloproteinase involved in extracellular matrix degradation and metastatic dissemination, together with increased CDK2 (1405_i_at) expression, a key regulator of G1/S cell cycle transition. This combination suggests a transcriptional profile consistent with proliferative and invasive tumor behavior, potentially reflecting a more aggressive phenotype.

Cluster 0 demonstrated higher expression of *CXCL8* (1553970_s_at), a pro-inflammatory chemokine implicated in tumor-promoting inflammation, and *VEGFA* (1552348_at), a principal mediator of angiogenesis. This pattern is indicative of a microenvironmentally driven program characterized by inflammatory signaling and vascular remodeling, processes known to facilitate tumor progression and immune modulation.

Cluster 2 exhibited relatively increased expression of *TP53*-related transcripts (e.g., 1552309_a_at) and anti-apoptotic regulators such as B-cell CLL/lymphoma 2 (BCL2) (not shown in the top 10 heatmap but enriched within the cluster), suggesting a transcriptional configuration associated with enhanced cell survival and resistance to apoptosis. This subtype may represent tumors with preserved or reprogrammed stress-response mechanisms.

Collectively, these cluster-specific expression profiles support the existence of biologically distinct CRC subtypes driven by divergent oncogenic axes, including invasion/proliferation (Cluster 1), inflammation/angiogenesis (Cluster 0), and survival signaling (Cluster 2).

Importantly, this stratification reinforces the concept that CRC recurrence risk is likely mediated by coordinated pathway-level dysregula tion rather than isolated gene alterations. The identification of subtype-specific transcriptional programs may have prognostic implications and could inform targeted therapeutic strategies, particularly in the context of multimodal integration with radiomic and, prospectively, renal function parameters.

The supervised classification analysis demonstrated that Random Forest achieved the highest predictive performance, with a test set AUC of 0.955 and an F1-score of 0.857, indicating excellent discriminative ability and a balanced trade-off between precision and recall. The high AUC suggests strong separation between recurrence and nonrecurrence classes, while the F1-score confirms stability in handling both false positives and false negatives within a moderately sized cohort.

Logistic regression ranked second in performance, achieving a precision of 0.833 and a robust cross-validated AUC (AUC CV) of 0.939, supporting its generalizability. Notably, the strong crossvalidation performance of this linear model suggests that the predictive signal captured by the selected gene set is structured and not solely dependent on non-linear interactions. In small-to-moderate sample settings, regularized linear models may offer improved stability compared to more complex ensemble approaches.

In contrast, XGBoost, decision Tree, and gradient boosting demonstrated inferior performance across multiple evaluation metrics (Table 3). This reduction in predictive accuracy likely reflects the combined effect of limited sample size and relatively high feature dimensionality, conditions under which high-capacity models are more prone to overfitting. The discrepancy between crossvalidation and test metrics in these models further supports this interpretation.

**TABLE 3.** Performance of tested classifier to predict disease-free survival (DFS)

Model	Accuracy	Precision	Recall	F1-score	AUC Test	AUC CV
Random forest	0.842	0.9	0.818	0.857	0.955	0.92
Logistic regression	0.842	0.833	0.909	0.87	0.909	0.939
Gradient boosting	0.789	0.818	0.818	0.818	0.818	0.703
XGBoost	0.684	0.778	0.636	0.7	0.818	0.878
Decision tree	0.632	0.7	0.636	0.667	0.631	0.616

1 AUC CV = area under the receiver operating characteristic curve cross-validated

**FIGURE 7.**
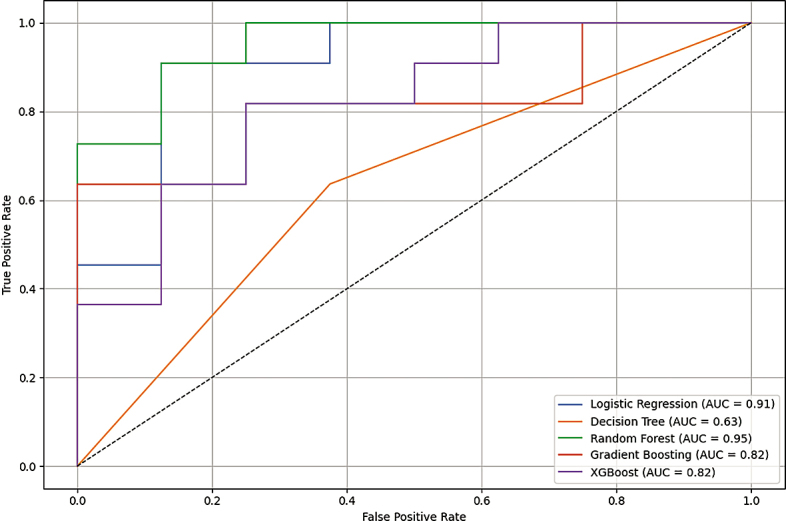
Receiver operating characteristic (ROC) curves of tested classifier to predict disease-free survival (DFS).

Receiver operating characteristic (ROC) curves confirmed the superior discriminative capacity of random forest and logistic regression (Figure 7), both demonstrating clear deviation from the diagonal reference line representing random classification. The consistent performance across threshold-independent (AUC) and threshold-dependent (F1-score, precision, recall) metrics reinforces the robustness of the predictive signal derived from the selected transcriptomic features.

Although these findings are derived from a modest cohort and should be interpreted as proof-of-concept, they demonstrate the feasibility of recurrence prediction using a reduced transcriptomic signature and provide a methodological foundation for future multimodal modeling in larger, prospectively collected datasets such as the SIRIO cohort.

**FIGURE 8.**
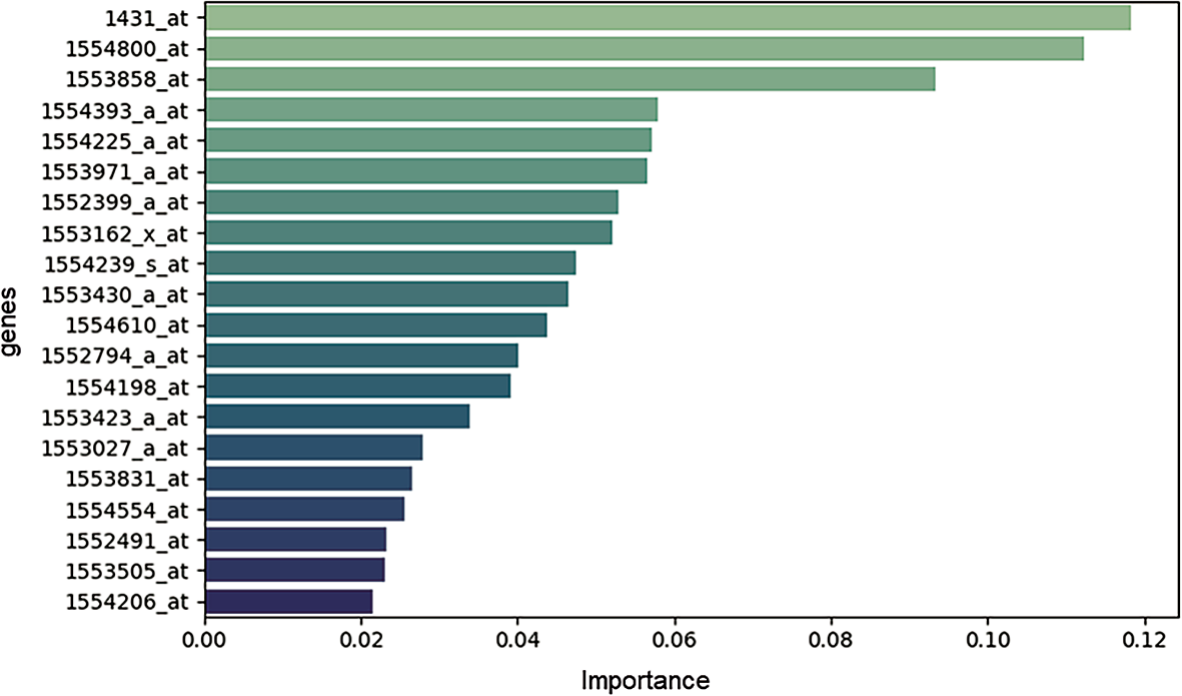
Feature importance analysis.

Lastly, the feature importance analysis derived from the Random Forest classifier (Figure 8) identified three transcripts as the most informative predictors of DFS event: 1431_at *(CYP2E1*-Cytochrome P450 family 2 subfamily E member 1), 1554800_at (*RAB39A*), and 1553858_at (*ZBTB3*-Zinc finger and BTB domain containing 3). These genes contributed most substantially to the model’s discrimina-tive performance, indicating a strong association with recurrence risk within this cohort.

*CYP2E1* encodes a member of the cytochrome P450 superfamily involved in xenobiotic metabolism and reactive oxygen species (ROS) generation. Its role in oxidative stress regulation may influence tumor progression through DNA damage accumulation, metabolic adaptation, and microenvironmental remodeling.

*RAB39A*, a member of the RAS oncogene family, is implicated in vesicular trafficking and intracellular signaling. Given the established relevance of RAS pathway alterations in colorectal cancer, its prominence in the feature importance ranking is biologically plausible and suggests a potential role in modulating proliferative or metastatic signaling networks.

*ZBTB3* is a transcriptional regulator belonging to the zinc-finger and BTB domain-containing protein family. Although less extensively characterized in CRC, it has been implicated in cellular differentiation, transcriptional repression, and tumor-associated signaling pathways, supporting its candidacy as a regulatory driver of recurrence-associated transcriptional programs.

Importantly, these genes do not belong to a single pathway but rather span metabolic regulation, oncogenic signaling, and transcriptional control. This heterogeneity reinforces the concept that recurrence risk is multifactorial and driven by coordinated dysregulation across biological domains rather than isolated molecular events.

While the current findings should be interpreted cautiously due to the limited sample size, the consistency of their importance within the ensemble model highlights *CYP2E1, RAB39A*, and *ZBTB3* as promising candidates for future molecular validation. Independent cohort replication, functional assays, and integration with radiomic and renal function parameters in prospective datasets such as the SIRIO study will be necessary to confirm their prognostic and mechanistic relevance.

## Discussion

In this study, we performed an integrative transcriptomic and machine learning analysis to identify molecular features associated with CRC recurrence. In parallel, we systematically evaluated publicly available datasets to investigate the intersection between CRC and CKD. Our findings revealed a critical and previously underappreciated limitation: the absence of structured datasets simultaneously capturing renal function parameters alongside multi-omic cancer data.

Emerging evidence indicates that CKD is not merely a comorbidity but may act as a biologically active modifier of carcinogenesis. Patients with CKD exhibit increased systemic inflammation, oxidative stress, immune dysregulation, endothelial dysfunction, and accumulation of uremic toxins – mechanisms that may collectively promote tumor initiation and progression. Epidemiological studies consistently report elevated incidence and mortality rates of CRC among CKD populations^25-28^, supporting the existence of a clinically relevant and biologically plausible CRC-CKD axis.

Despite this growing body of evidence, our evaluation of major oncologic repositories, including TCGA-COAD, cBioPortal, UCSC Xena Browser, and ICGC, demonstrated a striking lack of structured renal function data. Key variables such as eGFR, serum creatinine, CKD staging, or validated diagnostic codes were either absent or unusable. This limitation precludes direct investigation of how renal dysfunction may influence tumor genomics, transcriptomic programs, or recurrence dynamics. The fragmentation between nephrology datasets (which lack omics-level cancer data) and oncologic repositories (which lack renal metrics) represent a major barrier to mechanistic discovery.

Within the available transcriptomic dataset, our univariate analysis identified several genes significantly associated with DFS. Notably, *ALDH1A1* and *CDK2* were downregulated in patients experiencing DFS events, suggesting a context-dependent association with recurrence risk. While *ALDH1A1* has been described as a stemness-related marker linked to adverse prognosis in CRC^25^, its role may vary depending on molecular subtype and tumor microenvironmental context. Conversely, VEGFA and MMP9 were upregulated in the DFS-event group, consistent with their established roles in angiogenesis, extracellular matrix remodeling, invasion, and metastatic dissemination. These findings reinforce the central contribution of angiogenic and stromal remodeling pathways in recurrence biology.

Unsupervised clustering based on transcriptomic profiles identified three distinct molecular subgroups, with k = 3 supported by Elbow method, Silhouette score, and Gap Statistic. The resulting transcriptional signatures highlighted structured genomic heterogeneity within the cohort. This stratification aligns conceptually with the Consensus molecular subtypes (CMS) framework described by Guinney *et al*.29, which defines CRC subtypes based on immune activation, metabolic dysregulation, stromal enrichment, and genomic instability. Importantly, CMS subtypes have been shown to differ in prognosis and therapeutic responsiveness^30^, and their biological stability has been validated across preclinical and spatial transcriptomic studies.^31^

Although our clustering did not aim to formally replicate CMS classification, the emergence of three coherent transcriptomic subgroups supports the notion that intrinsic molecular architecture can be captured through gene expression profiling, even in modestly sized cohorts. This reinforces the clinical relevance of transcriptomic stratification as a foundation for precision oncology.

Supervised machine learning models further demonstrated the feasibility of recurrence prediction using a reduced gene signature. Random forest and logistic regression achieved the highest predictive performance (accuracy 0.842), with random forest reaching a test AUC of 0.955 and logistic regression demonstrating strong crossvalidated stability (AUC CV 0.939). The strong performance of a regularized linear model suggests that the predictive signal is structured and not exclusively dependent on complex nonlinear interactions. In contrast, boosting-based and tree-based high-capacity models exhibited reduced performance, likely reflecting overfitting risk in small, high-dimensional datasets.

Feature importance analysis identified *CYP2E1, RAB39A*, and *ZBTB3* as the most informative predictors of DFS events. *CYP2E1* is involved in oxidative stress and xenobiotic metabolism, processes particularly relevant in systemic inflammatory and uremic states. *RAB39A*, a member of the RAS oncogene family, aligns with established evidence linking RAS pathway dysregulation to CRC progression and radiogenomic signatures.^32-37^ *ZBTB3*, a transcriptional regulator, may contribute to recurrence-associated transcriptional reprogramming. The fact that these genes span oxidative stress, oncogenic signaling, and transcriptional regulation underscores the multifactorial nature of recurrence biology.

Importantly, several of the pathways emerging from our analysis – including inflammatory signaling (IL6/STAT3 axis), angiogenesis (VEGFA), extracellular remodeling (MMP9), and oxidative stress regulation (CYP2E1) are mechanistically plausible mediators of the CRC-CKD interplay. CKD-associated chronic inflammation and endothelial dysfunction may amplify these pathways, potentially altering tumor behavior and recurrence risk. However, due to the absence of renal function data in public repositories, these hypotheses cannot currently be tested in a multi omic framework.

Moreover, due to the limited availability of publicly accessible datasets containing both transcriptomic profiles and DFS information, external validation was not feasible in the present study and represents an important objective for future investigations.

Recent bibliometric analyses confirm that radiomics research in CRC is expanding rapidly, particularly in MRI and CT-based prediction of molecular alterations.^32-42^ Nevertheless, publicly available radiomic feature sets remain scarce, further compounding the challenge of integrative modeling. The convergence of these gaps, renal metrics absence, limited radiomic accessibility, and fragmented omics resources, highlights the urgent need for prospectively designed multimodal datasets.

The PNRR-funded SIRIO project (PNRR-MCNT1-2023-12378005)^5^ was conceived precisely to address this unmet need. SIRIO is a prospective, multicenter study designed to recruit CRC patients with and without CKD, integrating structured renal function parameters with radiomic, transcriptomic, and liquid biopsy data. Through harmonized imaging acquisition, centralized radiomic extraction, spatial transcriptomics, and machine learning-based integration, SIRIO aims to elucidate the biological mechanisms linking renal dysfunction to colorectal tumorigenesis and recurrence.

In conclusion, our study demonstrates the feasibility of transcriptomic-based recurrence modeling in CRC while simultaneously exposing a critical structural gap in existing public datasets: the absence of integrated renal and multi-omic cancer data. The identification of biologically coherent transcriptomic clusters and recurrence-associated genes provides a proof-of-concept framework. However, definitive characterization of the CRC-CKD axis will require dedicated prospective cohorts integrating renal, molecular, and imaging domains. The SIRIO initiative represents a strategic step toward closing this gap and advancing precision oncology at the intersection of nephrology and colorectal cancer researchs.
